# CAR-NKT cell therapy: a new promising paradigm of cancer immunotherapy

**DOI:** 10.1186/s12935-023-02923-9

**Published:** 2023-05-08

**Authors:** Kaveh Hadiloo, Safa Tahmasebi, Abdolreza Esmaeilzadeh

**Affiliations:** 1 Student Research Committee, Department of immunology, School of Medicine, Zanjan University of Medical Sciences, Zanjan, Iran; 2 Student Research Committee, Department of immunology, School of Medicine, Shahid beheshti University of Medical Sciences, Tehran, Iran; 3 Department of Immunology, Zanjan University of Medical Sciences, Zanjan, Iran; 4 Cancer Gene Therapy Research Center (CGRC), Zanjan University of Medical Sciences, Zanjan, Iran

**Keywords:** Natural killer T cell, Chimeric antigen receptor, CAR, Cancer, Immunotherapy

## Abstract

Today, cancer treatment is one of the fundamental problems facing clinicians and researchers worldwide. Efforts to find an excellent way to treat this illness continue, and new therapeutic strategies are developed quickly. Adoptive cell therapy (ACT) is a practical approach that has been emerged to improve clinical outcomes in cancer patients. In the ACT, one of the best ways to arm the immune cells against tumors is by employing chimeric antigen receptors (CARs) via genetic engineering. CAR equips cells to target specific antigens on tumor cells and selectively eradicate them. Researchers have achieved promising preclinical and clinical outcomes with different cells by using CARs. One of the potent immune cells that seems to be a good candidate for CAR-immune cell therapy is the Natural Killer-T (NKT) cell. NKT cells have multiple features that make them potent cells against tumors and would be a powerful replacement for T cells and natural killer (NK) cells. NKT cells are cytotoxic immune cells with various capabilities and no notable side effects on normal cells. The current study aimed to comprehensively provide the latest advances in CAR-NKT cell therapy for cancers.

## Introduction

Recently, chimeric antigen receptor (CAR)-armored cell therapy has focused on the T cell [1]. This cell has demonstrated outstanding capability against different tumors and can treat them appropriately [2], especially in hematological malignancies [3, 4]. Up to now, six FDA-approved CAR-T cell-based drugs have been developed for hematological malignancies, including Kymriah, Yescarta, Tecartus, Breyanzi, Abecma, and Carvykti [5]. Unlike their excellent advantages, they have several side effects and a high cost per dose that limit their use for clinical purposes [6, 7]. In continuation, scientists explored to find other potential immune cells as CAR-based cell therapy candidates. One of the useful cell candidates is NKT cells, a subset of the innate immune system that compose circulation cells [8]. NKT cells bridge the innate and adaptive immune systems by producing different biological factors and are considered potent cytotoxic cells against tumors [9].

NKT cells have a vital role in the upregulation of the immune system and suppression of the tumor microenvironment (TME) in the course of disease [10]. In addition, NKT cells have increased cytolytic activity with good infiltration ability into TME, which causes the tumor cell targeting to be powerful [11]. NKT cells have brilliant features that make them very useful cells against tumors, including double function capability (the ability to use innate receptors and engineered receptors for activation) [12], MHC-independent cytotoxicity, success in the “Off-The-Shelf” process (the rate of capability for creating the new modified cells), as well as improved anti-tumor immunity in the anergy process [13]. Some natural features of NKT cells convert these cells into powerful candidates, for instance, in the rapid detection and eradication of tumors, the elimination of cancer cells in early cell growth [14], and fighting tumors even during the immune system disability [15]. NKT cells can activate other immune cells by producing interleukin 2 (IL-2) and IL-21, which cause the activation of NK cells and T cells [16]. The CAR structure, when added to the NKT cell, creates a powerful cell with different cytotoxic features against cancers.

The current study aimed to comprehensively discuss the various aspects of CAR-NKT cell therapy and its recent advances in cancer treatment. The different advantages of this treatment, the comparison with other CAR-armored immune cells, and the related preclinical and clinical findings of cancer CAR-NKT cell therapy have been discussed in detail. Also, the limitations, challenges, and solutions for increasing the safety and effectiveness of CAR-NKT cell treatment have been addressed to arrive at the conclusion that CAR-NKT cells whether can be effective in cancer treatment.

## NKT cells

### Types and structure

NKT cells are divided into three types that have remarkable properties listed in Table 1. The most famous type of NKT cells is type I, namely invariant NKT (iNKT) cell. This cell is restricted to T-cell receptor (TCR) and expresses a semi-invariant TCR-α chain due to the limited TCR source [17]. Since the iNKT cell continues to express factors that are dependent on T cells for maturation, like CD4 and CD8, this cell type is divided into three subtypes; CD8^+^, CD4^+^, and double-negative (CD4^− ^CD8^−^ or DN) cells [18]. CD8^+^ and DN cell types can produce Th1 cytokines, while the production of Th2 cytokines like IL-4 and IL-13 is unique to the CD4^+^ cell type. CD4^+^ cells are considered helper or regulatory cells, while CD8^+^ and DN cells are considered effector cells with cytotoxic properties [10]. Among these types, CD8^+^ cells have a more cytotoxic function and indicate upregulated expression of cytotoxic cell-associated receptors like CD16, NKG2C, and NKG2D [18, 19]. The delegate receptor of all these cells is α-galactosylceramide (α-GalCer) [20]. These subtypes differ in cytokine secretion profiles and the expression of chemokine receptors, integrins, and superficial receptors [21]. Type II NKT cells can strengthen cancers by activating myeloid-derived suppressor cells (MDSCs) and tumor-related factors in TME [17]. Since this type has no particular marker or agonistic antigen, its classification has different challenges. These cells have a more diverse repertoire of Va rearrangements like TRAV7, TRAV9, and TRAV12 that make them potent for easy recognition of sulfatide-ligands such as lysophosphatidylcholine [22]. Inhibition of these cell types may improve the function of effector NKT cells in the cancer treatment process [23].

**Table 1:** Comparing three main subtypes of NKT cells [8, 220, 221]

Special	Type 1 NKT	Type 2 NKT	NKT like cell
CD1d dependent	Yes	Yes	No
α-GalCer reactive	Yes	No	No
TCRa chain	Va14-ja18(mice), va24 ja18(human)	Diverse, but some vas3.2-ja9, Va8(mice)	Diverse
NK1.1 or CD161	Positive (resting mature)	Positive and negative	Positive
Restriction	CD1d	CD1d	MHC
Subset	CD4^+^DN(mice) CD4^+^CD8^+^DN(human)	CD4^+^ and DN	CD4^+^, DN, and CD8^+^
IL-4 production	Yes	Yes	No
IFNγ production	Yes	Yes	Yes
Development requirements	IL-15	Not Established	Not Established
Tissue location	Liver, spleen, intestine, and pulmonary mucosa	Liver and spleen	Liver, spleen, and bone marrow
TCRB chain	VB2, VB7, VB8.2 (mice) VB11 (human)	Diverse, but some VB8.2 (mice)	Diverse

### Activation and function

NKT cells are activated in different manners: (1) like T cells, by TCR and recognition of CD1d [24]; (2) like NK cell, by expressing killer cell immunoglobulin-like receptors (KIRs) and natural killer cell receptors (NLRs) [25, 26]; and (3) by cytokines like IL-12, IL-18, and interferon-γ (IFN-γ) [27]. The cytotoxicity and amount of cytokine reaction in NKT cells depend on factors such as glycolipid antigens, the subtype of NKT cells, and the tissue location of NKT cells [28]. The α-GalCer is a synthetic glycolipid discovered in a sponge, so-called Agelas mauritianus, that can bind to TCR and other receptors [29]. The α-galactosylceramide (α-GalCer) ligand activates the NKT cells by binding to CD1d receptors on antigen-presenting cells (APCs) [30]. NKT cell activation by α-GalCer has some beneficial points, including the rehabilitation of NK- and T-cells in the TME and recruiting their power in targeting the CD1d^+^ tumor cells [31], the rapid secretion of a wide range of cytokines [32], and double impact function.

Although α-GalCer has a potent anti-tumor effect on NKT cells, its repeated administration causes anergy [33]. Accordingly, chronic activation by α-GalCer downregulates the expression of TCR and NLR, increases the expression of inhibitory molecules like programmed cell death protein 1 (PD-1) and B- and T-lymphocyte attenuator (BTLA), terminates the activation-induced cell death process (AICD), decreases the production of IFN-γ and the IFN-c mRNA expression in anergic cells, increases the IL-4 mRNA expression, and induces the iNKT cell shift toward Th2 and T_reg_ cells [34]. This is an auto-regularity mechanism that NKT cells employ to prevent tissue damage [35], but in cancer patients, it causes tumor cell immune escape [36].

Another importent connection factor is the CD1d receptor, a non-polymorphic, glycolipid-presenting type of MHC class-1 like cell that is expressed in APCs and thymocytes [37]. The CD1 family is divided into several groups, among which CD1d is widely expressed in different cancers [38]. The CD1d^+^ tumors could be killed by NKT cells directly by recognizing CD1d-specific antigens on tumor cells; however, the clearance of CD1d^−^ tumors is mediated by other immune cells like NK- and T-cells [39].

The NKT cells can kill tumor cells without dependency on MHC but indirectly, they can target the MHC^+^ tumor cells by activating the CD8^+^ cells. In addition, other actions of NKT cells that induce tumor cell death include the stimulation of CD1d, activation of cell death receptors, and induction of perforin, granzyme B, and tumor necrosis factor-a-related apoptosis-inducing ligand (TRAIL) production [40]. One of the other highlights is the communication between NKT cells and DCs [41]. In advanced cancers, the DC cells cannot activate NKT cells but can express the α-GalCer antigen [42]. NKT cells activate the DC cells by affecting α-GalCer, strengthening the immune system response against cancer cells, and mediating the interactions between TCR and CD1d, as well as CD40L and CD40 [43]. On the one hand, activated DC cells can produce IL-12 and increase IFN-γ levels, which subsequently activate NK-, NKT-, and T-cells. Besides, DC cells express CD27L, CD70, and NKG2DL, which increase the cytotoxicity of NKT cells. On the other hand, NKT cells can stimulate the secretion of C-C motif chemokine ligand 17 (CCL17) by activating CD8α^+^ DC cells and attracting CD8^+^ cells to the tumor site [44].

NKT cells secrete large amounts of cytokines and chemokines, such as those regulated upon activation of normal T cell and presumably secreted (RANTES), Eoxtaxin, and macrophage inflammatory proteins-1a (MIP-1a). These chemokines improve the function of the immune cells and attract them to TME [45]. NKT cells also contribute to the function of immune system by suppressing the immunosuppressive cells in TME. For example, NKT cell type I can mediate anti-tumor activity in primary human neuroblastoma by inhibiting tumor-associated macrophages (TAMs), the immune suppressors that increase tumor progression [46, 47]. Besides, in an influenza type A virus model, it has been reported that NKT cell type I could inhibit MDSCs, the immune suppressive cells from the myeloid population [48].

### Comparison

Phenotypically, NKT cells have common markers with conventional T cells like TCR, CD4, and CD8, as well as NK cells like KN1.1, CD16, and CD56. Unlike conventional T cells, NKT cells express a limited range of TCR-α and β-chains and recognize CD1d on the APC cells instead of MHC. Like NK cells unlike T cells, NKT cells gain functional properties like producing cytokines during maturation and rapidly responding during antigen exposure [49]. Like conventional effector T cells, NKT cells are divided into three types (NKT-1, 2, and 17) and create related cytokines like IFN-γ, IL-4, and IL-17. Generally, NKT cells have three groups of receptors, including CD1d, the semi-I TCR-β chain, and iTCR [40]. NKT cells regulate the immune system by CD1d and cytokine secretion, but the T- and NK-cells do this only by cytokine secretion. Interestingly, due to the extensive expression of CD1d throughout the body, the NKT cell can regulate the immune response better than other immune cells [50].

Both NKT- and T-cells are derived from double-positive αβ T cells by different thymic selections and have a similar maturation process. However, aside from the T cells chosen with thymus epithelial cells, the NKT cells were selected from CD1d-expressing double-positive (CD4^+^ CD8^+^ or DP) thymocytes. Another difference is that the T cell is a member of the cellular immune system with more powerful cytotoxicity, while the NKT cell is a part of the innate immune system. Also, T cells have better transfection ability than NKT cells. However, the NKT cells have more genes related to the chemokines than T cells, suggesting that NKT cells may functionally regulate the immune system by improving chemokine-related interactions [51, 52].

In comparison between NKT- and NK-cells, the NKT cell is the type of T cell, but the NK cell is a member of the large granular lymphocytes. Also, NKT cell maturation occurs in the thymus and possesses TCR, but NK cell maturation occurs most in the blood circulation and possesses the F_c_ receptor along with activatory and inhibitory receptors. NKT- and NK-cells are the first-line cells involved in defensive responses in TME. In addition, the CAR structure of NK- and NKT-cells can be used along with intracellular signaling, but the T cell cannot use both signaling ways. In the last case, NKT cells do not contain cytoplasmic granules, but NK cells do contain cytoplasmic granules [53, 54].

## CAR-NKT cell, a potent player for cancer immunotherapy

Due to some barriers, such as the low frequency of NKT cells, insufficient infiltration into TME, and the majority of the CD1d^−^ tumor cell population, NKT cells cannot take on an efficient function in cancer treatment [55]. Nevertheless, because of the perfect brilliance of NKT cells and acceptable gene-engineering modification that makes them sinewy, combining NKT cells with CAR structures can create a potent effector cell that can elicit promising outcomes in cancer treatment [16].

The CAR structure enables the immune cells to respond to the specific antigens, selectively [61], regulates the activation of the armored cells, and provides MHC-independent antigen recognition [56]. CAR paves the way for recognizing a broad range of antigens like glycosylated proteins, glycolipids, carbohydrates, and conformational epitopes [57].

The CAR structure consists of three parts from the outside to the inside: the ectodomain, the transmembrane domain, and the endodomain. The ectodomain part is responsible for antigen recognition mediated by a single-chain variable segment (ScFv) structure. ScFv is connected to the transmembrane part by the spacer (hinge) region and is known as the most common structure for the ectodomain part. Its affinity affects the specificity of the CAR structure [58]. The spacer also has an essential role in the specificity of CARs. A short hinge has a better anti-tumor function, and a longer spacer seems to be more effective in targeting antigens in distal membrane cells [59].

The trans-membrane domain fixes the structure into the membrane, transfers the signals, and can comprise different molecules like CD3ζ, CD4, CD8, and CD28. The endodomain part is responsible for stimulating the CAR immune cells. The intracellular domain of CAR includes CD3ζ, which has a role in signal transfer. The co-stimulatory domains such as 4-1BB, CD27, CD28, or OX40 that can exist in the intracellular domain help to boost CAR cytolytic function, cytokine production, and persistence [60]. The NKT cell does not have a special co-receptor; instead, the CD4 molecule functions as a co-receptor [61]. The CAR function depends on the endodomain part, and any changes in this part can stimulate various signaling cascades and create diverse responses against target antigens. Some studies on the function of CAR-NKT cell co-stimulators demonstrated inaccurate results in these cells. For example, unlike CAR-T cells, in CAR-NKT cells, CD28 was suitable for proliferation, tumor-killing function, and survival. Furthermore, CD28 compels CAR-NKT cells for copious expansion, while 4-1BB induces excess activation and redirects them to cell death [62, 63].

**Fig. 1:**
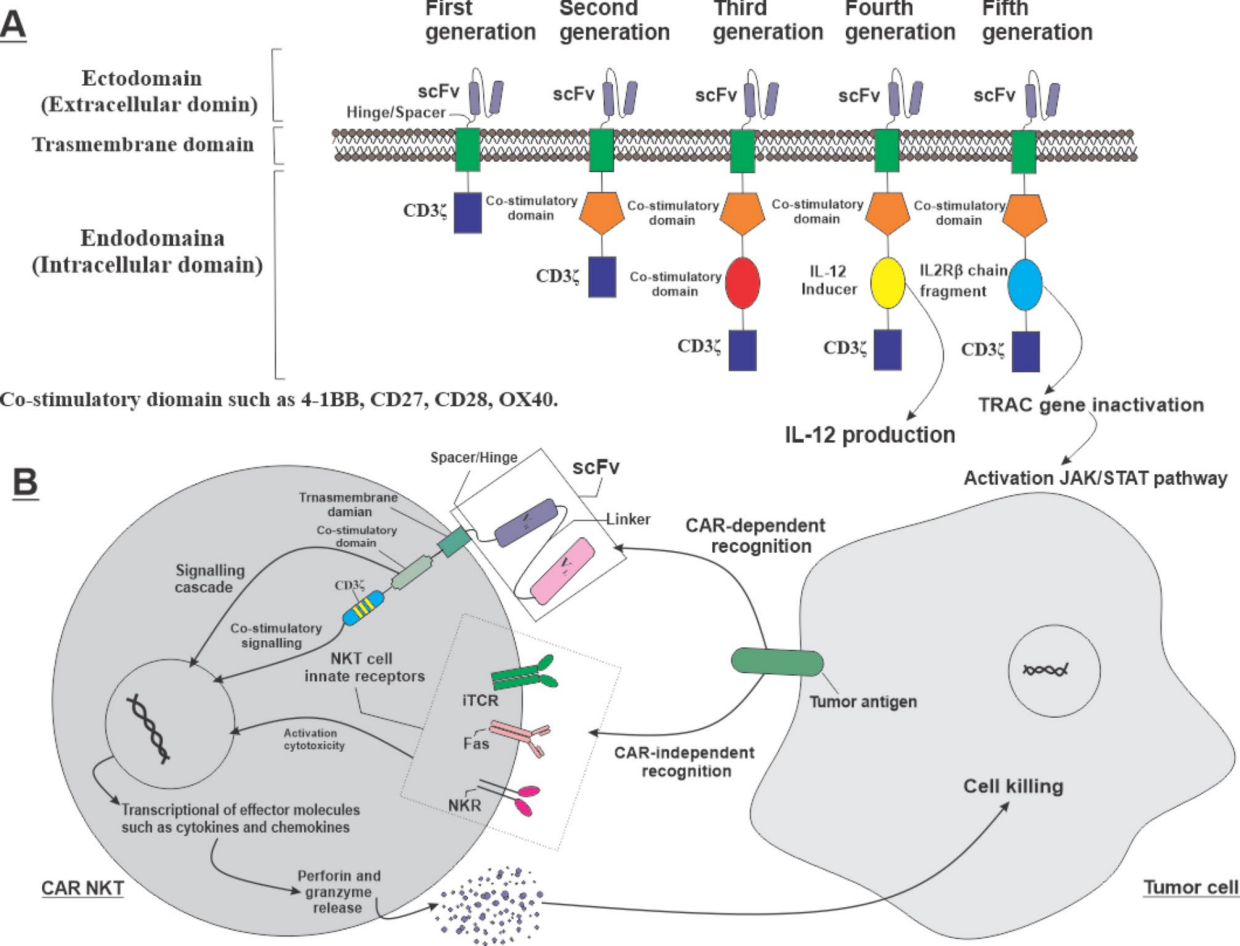
**A.The various generations of CAR structure**. The base structure comprises the ectodomain, transmembrane domain, and CD3ζ, which are shared among all generations. The main differences are related to the co-stimulatory domain and the related signaling cascades. Changes in each part of the structure can create new CARs with different features. **B.The function ofarmored **NKT cell **with CAR-structure**. The NKT cell can utilize CAR-based recognition, innate activatory receptor-based recognition, and the relevance of the “double-function” for better anti-cancer cytotoxicity.

The CAR structure divides into five generations based on operative molecular structure and costimulatory molecule existence (Fig. 1). The first generation is composed of ScFv and CD3ζ without co-stimulators [58]. The second and third generations of CAR have one and two costimulatory molecules, respectively (4-1BB, CD27, or OX40), in addition to ScFv and CD3ζ. The fourth generation of CAR, known as T-cells redirected towards universal cytokine killing (TRUCKs), is engineered by transducing transgenes for cytokine secretion (e.g., IL-12) or other biological factors. This type of CAR includes a nuclear factor of activated T cells (NFAT) that enables the cell to secrete cytokines. TRUCKs have shown excellent preclinical and clinical outcomes in cancer treatment [64]. The fifth generation of CAR has been recently introduced, possessing the additional co-stimulator domain to activate the new signaling pathways. In this structure, a co-stimulator is added to an intracellular domain of a cytokine receptor (e.g., IL2Rβ chain fragment) that connects with the STAT and CD27 domains. With this new structure, the CAR-T cells can induce the antigen-dependent activation of the JAK-STAT pathway, improve cell proliferation, and prevent terminal differentiation. Also, this structure has demonstrated better results in persistence and therapeutic effects in leukemia compared to prior generations [65].

**Fig. 2:**
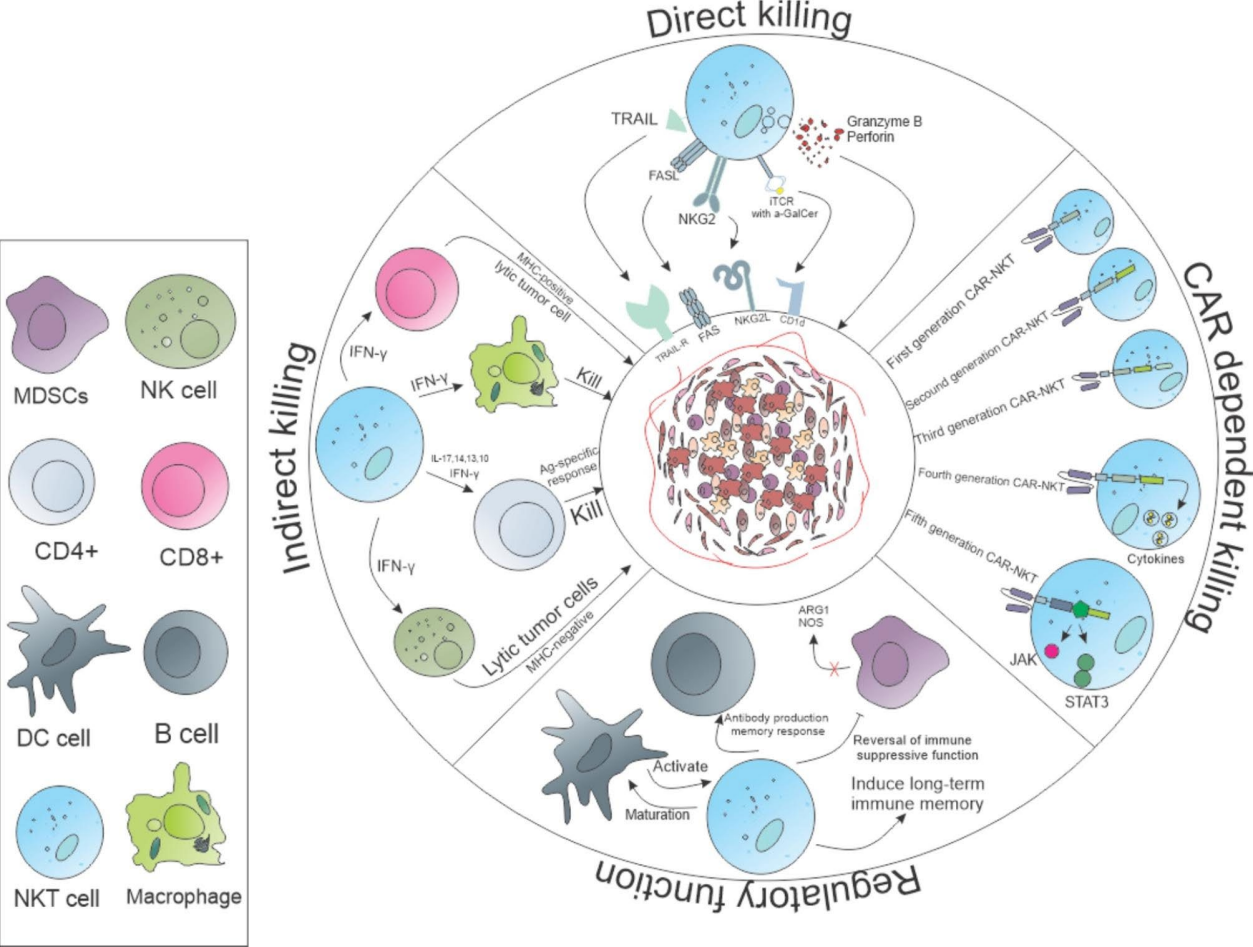
The different interactions of CAR-NKT cells with other immune cells and tumor cells. The CAR-NKT cell, by various methods, can regulate the immune system and lytic the tumors by or not CAR. Also, CAR-NKT cell can activate other immune cells like CD8-, CD4-, and NK-cells, and macrophages-. For instance, CAR-NKT cells can change the immune suppressive macrophages into immune-stimulating macrophages, and by increasing perforin and granzyme B, improve tumor eradication.

By transducing CAR structure into the NKT cell, the CAR-NKT cell has been created as a novel genetically-engineered cell with different abilities that can affect the cancer cells differently. Thevarious actions of these cells against tumors have been demonstrated in Fig. 2. The CAR-NKT cell can destroy tumor cells by a direct mechanism with its innate receptors and also by influencing other immune cells indirectly [40], which is caused by the increased specificity of the CAR structure. Interestingly, the CAR-NKT cell plays an essential role in cancer treatment by regulating the immune system, inhibiting some immunosuppressive cells, and empowering cellular immune responses. CAR-NKT cells, like CAR-T cells, obtained encouraging results in treating non-solid tumors in functional, *in vitro* and *in vivo* studies [66]. However, CAR-NKT cells coruscate more in solid tumors due to their unique features, like high infiltration cells into TME with low anergy. Promising outcomes have been reported in CAR-NKT cell therapy for solid tumors [6].

Furthermore, the CAR-NKT cell does not burden significant side effects, and their safety has been proven previously [67]. The other significant advantage of CAR-NKT cells will be their low final price due to their limited complexity, low need to repeat administration, no personal dependency due to MHC-independence, and limited occurence rate of Graft Versus Host Disease (GVHD) toxicity. Until now, the second and third generations of CAR-NKT cells have been developed. As another benefit,  it has also been proven that the little alterations in CAR-NKT cell structure, like the change in costimulatory molecules, create variations in the cytokine secretion profile. Thereby, due to the possibility of acquiring various CAR structures, the CAR-NKT cell can havea wide range of functions in cancer treatment [68]. Consequently, the CAR-NKT cell can be recommended as a potent therapeutic cell in cancer, with effective capabilities that can positively affect the success of cancer treatment in the future.

## CAR-NKT cell manufacturing

The process of CAR-NKT cell manufacturing is like CAR-T- and CAR-NK-cells, but with some differences. The manufacturing process included cell collection, activation, expansion, transduction (CAR-gene delivery into the cell), and reinfusion of CAR-NKT cells into the patient, intravenously or intratumorally.

To obtain cell sources, autologous and allogeneic NKT cells can be collected from cancer patients and healthy individuals, respectively. The NKT cells have distinct sources with various features. For instance, hematopoietic stem cell-engineered iNKT cells (HSC-iNKT) can effectively treat hematologic cancers in mouse models. They can be collected from umbilical cord blood (UCB), bone marrow (BM), and PBMCs [69]. HSC-iNKT cells are a promising source for engineering CAR-NKT cells and provide efficient treatment in patients with resistance to high doses of chemotherapy and radiotherapy [70]. As another example, HSC cells are an excellent source for producing human allogeneic HSC-engineered iNKT cells (^Allo^HSC-iNKT) with high purity and efficiency that can be engineered with CAR structure. ^Allo^HSC-iNKT cells are a good candidate for off-the-shelf CARs due to their lack of significant toxicities and because they may resist allo-rejection due to their unique features. Also, making HLA-ablated universal HSC-iNKT cells and their derivatives, like CAR-iNKT cells, may improve the persistence and anti-tumor function of these cells *in vivo* [71].

The process of cell isolation and expansion is essential due to the small number of NKT cells in peripheral blood circulation. Different approaches can be used to expand NKT cells. NKT cell collection from different sources may be efficient; for example, NKT cells from PBMCs present more α-GalCer and agonists of IL-2 [72]. Exley et al. suggested an approach that relies on cell selection based on polyclonal and monoclonal antibodies (mAbs) that reactive with human CD1d- invariant T cells (mAb6B11), followed by activation and expansion with glycolipid compounds via APC CD1d^+^ cells [73]. They successfully used unspecific mitogens like activating anti-CD3 mAb and created cell lines in 2–3 weeks. Nevertheless, their application is limited to preparing high-purity NKT cells based on clones propagated by α-GalCer CD1d in previous rounds [74, 75].

After activation, CAR gene transduction into the NKT cell should be done with the help of growth factors and vectors. Today, the experience of isolation, transduction, and expansion of CAR-NKT cells is limited and needs more investigation. One of the essential ligands in this field is CD62L, which can induce expansion in CAR-NKT cells. The CD62L^+^ and CD62L^-^ cells have no differences in cytolytic function or cytokine secretion, but in terms of persistence and proliferation, they are different [76]. Additionally, IL-21 can selectively protect CD62L^+^ NKT cells and improve their function in CAR-based immunotherapy [77]. IL-21 selectively inhibits the expression of BIM (pro-apoptotic factor) in CD62L^+^ cells without any effect on the expression of BCL2 (anti-apoptotic factor) and protects cells against AICD [77]. Karadimitris et al. optimized a protocol that offers a substantial expansion of CAR-19-engineered NKT cells from all sources, which can be used in the shelf setting against CD1d^+^/CD19^+^ expressing lymphoid tissues. Upfront expansion of NKT cells and lentiviral transduction of CD3/CD28-activated NKT cells in the presence of autologous APC and IL-15 build an active source of cells [78]. Simon et al. accessed NKT cells and CD8^+ ^T cells from the PBMC of healthy donors and stimulated this population of cells with OKT-3 and IL-2. They detected that both cell lines were expanded well, while the percent of NKT cells was lower than that of CD8^+^ T cells [79]. Anyhow, the manufacturing of CAR-NKT cells needs more experience and time to find new ways and better processing approaches. However, the existing methods have more off-the-shelf potential and lower costs than CAR-T cell production.

## CAR-NKT- vs. CAR-T- and CAR-NK-cells for cancer immunotherapy

The CAR-NKT cell has several advantages compared to CAR-T- and CAR-NK-cells and has been introduced as a better approachto cancer treatment. Based on this, a comprehensive comparison of various aspects has been made between the mentioned CAR immune cells, which have been listed in Table 2. Despite the encouraging outcomes of cancer treatment with CAR-T cells, the side effects of this treatmentare an ongoing problem [80]. CAR-T cells have different adverse effects that sometimes cause death in patients and burden the healthcare system and patients at a high cost [81]. Interestingly, most studies have reported that CAR-NKT cells have no obvious side effects and recommended that treatmentwith CAR-NKT cell would be safe. The reported adverse effects related to CAR-NK cell therapy are rare, as well. In a case report, severe side effects like cytokine release syndrome (CRS) have been reported after CAR-NK cell administration in non-small cell lung cancer patients [82]. CRS, also known as a cytokine storm or cytokine-associated toxicity, is brought on when the immune system overreacts to an infection like COVID-19 or following immunotherapy treatments like CAR immune-Cell therapy by producing a large number of inflammatory cytokines, especially IL-6 [83–85].

**Table 2:** The comparison between the different CAR-armed cells

Variable	CAR-T cells	CAR-NK cells	CAR-NKT cells	Ref.
Features/ Population	• Express TCR. • Heterogeneous. • Cellular-immune system. • MHC-I/II-restricted. • Thymus-dependent.	• Lack TCR. • Homogenous. • Innate immune system. • Non-MHC restricted. • Has NCR (KIR and NKGD2).NK1.1^+^. • Thymus independent.	• Express αβTCR and NK cell markers. • Homogenous. • Innate immune system. • Recognize α-GalCer. • Non-MHC restricted. • TCR & CD1d restricted. • Thymus-dependent.	[8, 95, 101]
Cell sources	• Autologous PBMCs. • UCB (rarely). • iPSCs.	• PBMCs. • UCB. • hPSC/iPSCs. • NK cells line.	• PBMCs. • UCB. • hPSC/iPSCs.	[102, 103]
Function	Effector	Effector	Adjuvant	-
Preparation/ expansion	• Active with anti-CD3/CD28 beads. • Do not need to feeder cells.	• Enrichment magnetic T-cell depletion CD56^+^ selection. • Using of IL-2. • Do not need to feeder cell.	• Activation with α-GalCer-pulsed PBMCs. • Using of IL-2.	[104, 105]
Activation and cytotoxic manner	• TCR manages processed antigens with MHC content in intra-cellular. • Complete activation by costimulatory signals. • Inducing apoptosis.	• Performance by ADCC, perforin, and granzyme secretion. • Expression apoptosis-inducing ligands. • Cytotoxic function regulates by stimulatory and inhibitory signals. • Cytotoxicity without sensitization or HLA-matching.	• Ability to recognize and destroy CD1d^+^ cells. • Regulate the immune system and activation of other cells. • Activation is required for costimulatory signals.	[75, 106]
CARs action	• When armed with CAR, only work CAR-dependent. • Useless in losing tumor antigen.	• Cytotoxic property by dependent and independent CAR. • Activation by CAR without requiring stimulator/inhibitor signals.	• Cytotoxicity dependent and independent-CAR. • The indirect anti-tumor function.	[4, 107]
Side effects	• On target-off tumor. • CRS. • GVHD. • Neurotoxicity. • TLS.	• Lower GVHD. • Lower CRS. • Fever and fatigue.	• Maybe limit cytotoxicity ability. • Not reported anything in interim reports.	[108, 109]
Suicide gene required	Required for limiting on-target off-tumor.	Not be required.	Not be required commonly.	[67]
Pre/clinical functions	Very functional in pre/clinical evaluation.	Very functional in pre/clinical evaluation.	Very functional in pre/clinical evaluation.	[15, 95]
Off-the-shelf approach	Autologous cells needed the development of an allogeneic approach.	Potential off-the-shelf CAR-cell products.	Potential off-the-shelf CAR-cell products.	[110]
Primary Location	Tissue or blood.	Blood.	Tissue or blood.	[111]
Proliferation post-activation	Yes	No	Yes	
Memory/ Persistence	Yes (αβ T)	No	Yes	[111]

Similarly, some clinical outcomes showed the accompaniment of CAR-NK cells with some side effects, but CAR-NKT cells did not have any sign of them. However, the absolute resultsof CAR-NKT cell safety need more investigation [86]. A study on children with neuroblastoma cancer showed no CRS or neurotoxicity when administering the first or second-generation of CAR-NKT cells (NCT032294954). Additionally, CAR-NKT cells, due to the MHC-independent antigen recognition ability, the strange nature of TCR, and the non-production of IL-6, can prevent GVHD without increasing the risk of disease recurrence. Interestingly, transferred donor NKT cells were at least ten times stronger than T_regs_ in protecting animal models against GVHD, compromising the graft-versus-leukemia effect [87].

Regarding the cost of the therapy, the final cost of the CAR-NKT cells in the clinics is estimated to be much lower than CAR-T cell therapy. The high cost of CAR-T cell therapy is derived from adverse effects, side effect management, personal production, and repeated doses of prescription, while the CAR-NKT cell has none [88]. Regarding function, the CAR-NKT cell is ahead in migration to the tumor site as the first cell. Also, it has better infiltration into TME than other cells, so that it may have an earlier and more efficient function on tumor cells. A common advantage of CAR-NKT- and CAR-NK-cells is their double function. It means they can recognize tumor antigens by their receptors and CAR structure, while CAR-T cells only recognize them by their CAR structure. This advantage is predominant in CAR-NKT cells due to the broad expression of CD1d in different cells [89]. As another advantage, CAR-NKT cells allow DC cells to start a long-term anti-tumor function with T cells by inducing CD8^+^ T cell cross-priming with CD103^+^ CD8α licensing in murine models [44, 90–92]. In addition to one of the administration properties, CAR-T cells remain in the body for a month to a year, which can cause “on-target/off-tumor” toxicity. The comparative study by Rotolo et al. showed that CD1d-restricted engineered CD19-CAR-NKT cells acted more effectively than CD19-CAR-T cells against CD1d-expressing lymphomas *in vivo*. They did not see any trace of GVHD in mouse models and, interestingly, saw that only one dose of CAR-NKT cells was enough to affect tumor clearance, dramatically [14].

CAR-NKT cells also have several advantages in comparison with CAR-NK cells. Indeed, CAR-NKT cells have various receptors that can regulate the immune system better than NK cells. Also, unlike NK cells that attend to blood circulation, NKT cells exist in both blood and tissues, which is essential in solid tumors. Moreover, NKT cells have memory cells and more persistence than NK cells [93].

The other crucial excellence of CAR-NKT cells is their ability to eliminate solid tumors. The solid tumor complex structure makes a strong barrier to different treatments, but the results for CAR-NKT cells are hopeful. Unfortunately, the comparison between CAR-T- and CAR-NKT-cells has not been made in solid tumors, but the comparison in other cancer types showed better results of CAR-NKT cell therapies. In comparison with CAR19-T cells, CAR19-NKT cells, exhibited more effective tumor suppression with a higher penetration rate into the CNS and a greater survival rate [12]. In a study on melanoma, the chondroitin sulfate proteoglycan 4 (CSPG4) antigen was targeted as an essential tumor antigen. The findings of this study reported that CAR expression with an equal expansion rate was faster in CAR-NKT cells, while the number of T cells was ten times greater than NKT cells in PBMC. Compared to cytokine secretion, CAR-T cells produce more cytokines, but the cytokine secretion rate was significantly higher in both CAR-T- and CAR-NKT-cells; even with this, the CAR-NKT cell eliminated tumor cells faster [79]. Interestingly, the CAR cytotoxicity is almost equal between CAR-NKT- and CAR-T-cells, while the CAR-NKT cell has no more noticeable side effects than CAR-T cells [94]. Although research on CAR-NKT cells has not made significant progress, what has been done so far shows the high efficiency and safety of these cells with the least potential risk in cancer treatment.

## CAR-NKT cells in preclinical studies

Preclinical studies have been performed to evaluate the anti-tumor efficacy of CAR-NKT cells in different types of cancers *(*Table 3*)*. Haczey et al. were the pioneers in work on CAR-NKT cells. They transfected cells with retroviral targeting disialoganglioside (GD2), a prominent target antigen in neuroblastoma cancer cells. They created different types of CAR-redirected GD2-NKT cell constructs that encoded: (1) the CD3ζ chain alone, (2) CD3ζ with 4-1BB, (3) CD3ζ with CD28, and (4) CD3ζ with using CD28 and 4-1BB simultaneously as costimulatory endodomains. The expression of both CD28 and 4-1BB endodomains showed significantly enhanced persistence of NKT cells *in vivo*. All structures were effectively localized in the tumor site, appeared very cytotoxic against neuroblastoma expressing GD2 and CD1d^+^ inhibitory macrophages with promising outcomes, and indicated no evidence of GVHD [95]. Also, CAR-NKT cells improved treatment quality and increased resistance and localization of NKT cells in the TME without any significant special toxicities in neuroblastoma [96]. On the other hand, it has been reported that CAR-NKT cells pulsed by α-GalCer could secrete perforin and exert intrinsic cytotoxicity in targeting the Jurkat cells (the human T-cell leukemia cell line expressing CD1d molecules). In contrast, the CD8^+ ^T cells showed unspecific background lysis [79]. As another example, it was found that the CD62L-CD19 CAR-NKT cell caused a complete tumor regression in B-cell lymphoma mouse models. When CD62L is combined with CD86, 4-1BB, and OX40L co-stimulators, it could significantly improve the clinical scale of the expansion of the NKT cells [97].

**Table 3:** The preclinical studies in CAR NKT cells

Target	Generation	Cancer	Expansion	Activation	Costimulator	Ref.
GD2	Second/third	Neuroblastoma	IL-2	Feeder cell with α-GalCer	CD28/ 4-1BB/ both	[235]
GD2	Second/fourth	Neuroblastoma	IL-2,7,21	Irritated auto NKT-negative cell with α-GalCer	CD28/ 4-1BB	[63]
CD19	Second	B-cell lymphoma	IL-2	Irritated auto NKT-negative cell with α-GalCer	4-1BB	[97]
CD19	Second	B-cell lymphoma	IL-2,7,21	Irritated auto NKT-negative cell with α-GalCer	CD28/OX40-CD28	[77]
CD19	Second/third	B-cell lymphoma	IL-2 and 15	Irritated auto PBMC pulsed with CD38/28	4-1BB	[12]
CSPG4	Second	Melanoma	IL-2	Anti CD3 antibody	CD28	[236]
CD38/BMCA	Second	Multiple myeloma	IL-2,7,15	Irritated auto NKT-negative cell with α-GalCer	CD28/4-1BB	[237]
CD19, CEA, or HER2	Second	B-cell lymphoma	IL-2	cytokine-induced killer cells (CIKZ)	CD28	[98]

In another preclinical study, the gamma delta (γδ) T cell (Vy9Vdelta2) and cytokine-induced killer (CIK) cells have been expanded simultaneously, which are termed CIKZ cells [60]. Both cells are a heterogeneous population of T- and NKT-cells that have been separately expanded and have shown remarkable cytotoxicity against different tumor cells *ex vivo*. The expanded CIKZ cells showed anti-tumor cytotoxicity and could be modified to express anti-C19 CAR, anti-HER2 CAR, and anti-CEA CAR. The CAR-modified CIKZ cells illustrated a better cytotoxicity effect than CAR-modified αβ T cells. In addition, researchers reported the anti-tumor properties and superior survival following the administration of CD19-CIKZ-CAR NKT cells along with the CD28 endodomain in B-cell lymphoma mouse models [98].

Another investigation focused on the activity of CAR-NKT cells through host CD8^+^ T cell cross-priming and demonstrated that CD19.28z-CAR NKT cells have a dose-dependent cytotoxic effect on CD19-expressing luciferase-transduced A20-lymphoma cells. CAR-NKT cells could significantly control the progression and development of tumor cells and improve animal survival compared to untreated mice and those receiving untransduced NKT cells. The co-administration of allogeneic CAR-NKT cells and autologous CD8^+^ T cells had a synergic effect and could significantly improve tumor control and increase mouse model survival. Also, allogeneic CAR-NKT cells shaped the CD8^+^ T cell compartment phenotypically, transcriptomically, and clonally repertoire. These results showed that the allogeneic CAR-NKT cells were significantly better than allogenic conventional CAR-T cells at inducing extended tumor control in immunocompetent hosts [9].  Another recent preclinical study reported that the CD38 and plasma cell-specific B cell maturation antigen (BMCA) targeting CAR-NKT cells successfully eliminated multiple myeloma (MM) tumor cells without any TCR-dependent cytotoxic activity. This combination system produced many cytokines and vigorous expansion by stimulation [99].

In summary, all preclinical studies on the efficacy and safety of these cells have acknowledged that CAR-NKT cells are fully functional *in vitro* and *in vivo* and that their use is quite effective against cancer cells. Although there are few pre-clinical studies, the first results showed the high efficacy and safety of these cells against tumors.

## Clinical applications of CAR-NKT cells

Due to the promising results of CAR-NKT cells in preclinical studies, some clinical trials have also been developed to evaluate the safety and efficacy of CAR-NKT cell therapy in cancers; some have published results, but others are ongoing *(*Table 4*)*. For example, Heczey et al. conducted a trial study to evaluate the autologous CAR-GD2-expressing IL-15 NKT cells (GINAKIT2) on the refractory/relapsed neuroblastoma cancer in three child patients by CD4^+^ CD62L^+^ cells (NCT03294954). The results indicated CAR-NKT cells could be greatly expanded *in vivo*, localized in the tumor microenvironment, and eradicated cancer cells effectively. They demonstrated that GINAKIT2 had better tumor infiltration, persistence, and anti-tumor activity than GD2-CAR NKT cells *in vivo*. The stimulation of NKT cells was done by α-GalCer, and the end purity was incredible (94.7%). They administered dose resistance to patients entirely and did not observe any dose-limiting toxicity like CRS or neurotoxicity. In addition, this structure can be detected in the body and, after being administered to it,induce bone metastasis and tumor regression in relapsed or refractory disease [96].

**Table 4:** The CAR-NKT cells in the clinical trial

Number	NCT	Phase	Notes	Cancer Type	Number of patients	Date	Treatment	Country	Ref.
1	02439788	-	Withdrawn, replaced by: NCT032294954	Relapsed or refractory Neuroblastoma	-	2015	IC9-anti-GD2-CD28/OX40/CD3ζ CAR NKT (GINAKIT) cells.	USA	[113, 116]
2	03294954	I	Recruiting; new CAR construct	neuroblastoma	24	2018	Autologous Anti-GD2-CD28/CD3ζ-IL15 CAR NKT (GINAKIT2) cells.	USA	[15, 70, 104]
3	03774654	I	Recruiting	B cell malignancy	48	2020	Allogenic Anti-CD19-CD28/CD3ζ-IL15 CAR NKT (ANCHOR) cells.	USA	www.clinicaltrials.gov
4	04814004	I	Recruiting	ALL B cell lymphoma cell	20	2021	Autologous CD19-CAR NKT cell expressing IL-15.	China	www.clinicaltrials.gov
5	05487651	I	Not yet recruiting	Refractory/relapsed B-cell NHL or leukemia (ALL or CLL).	36	2022	Allogeneic NKT-Cell Expressing CD19 Specific CAR and IL-15 in Relapsed or Refractory B-Cell.	USA	www.clinicaltrials.gov

The second area of ongoing research has focused on the feasibility and safety of hCD19.IL15.CAR-NKT cells aim to eradicate relapsed/refractory high-risk B-cell tumors. The structure used in this investigation is universal NKT cell expression hCD19 CAR and IL-15 (NCT04814004). Recently, a trial study evaluated allogeneic NKT-cells expressing CD19-specific CAR and IL-15 in relapsed or refractory B-cell malignancies (NCT05487651). This multi-center study evaluates the safety of KUR-502 in subjects with refractory/relapsed B-cell NHL or leukemia. However, efforts to advance the use of CAR-iNKT cell cancer treatment are still ongoing, and more clinical trials are needed to prove the effectiveness of this treatment in different hematological and solid tumors.

## Challenges in CAR-NKT cell therapy

**Fig. 3:**
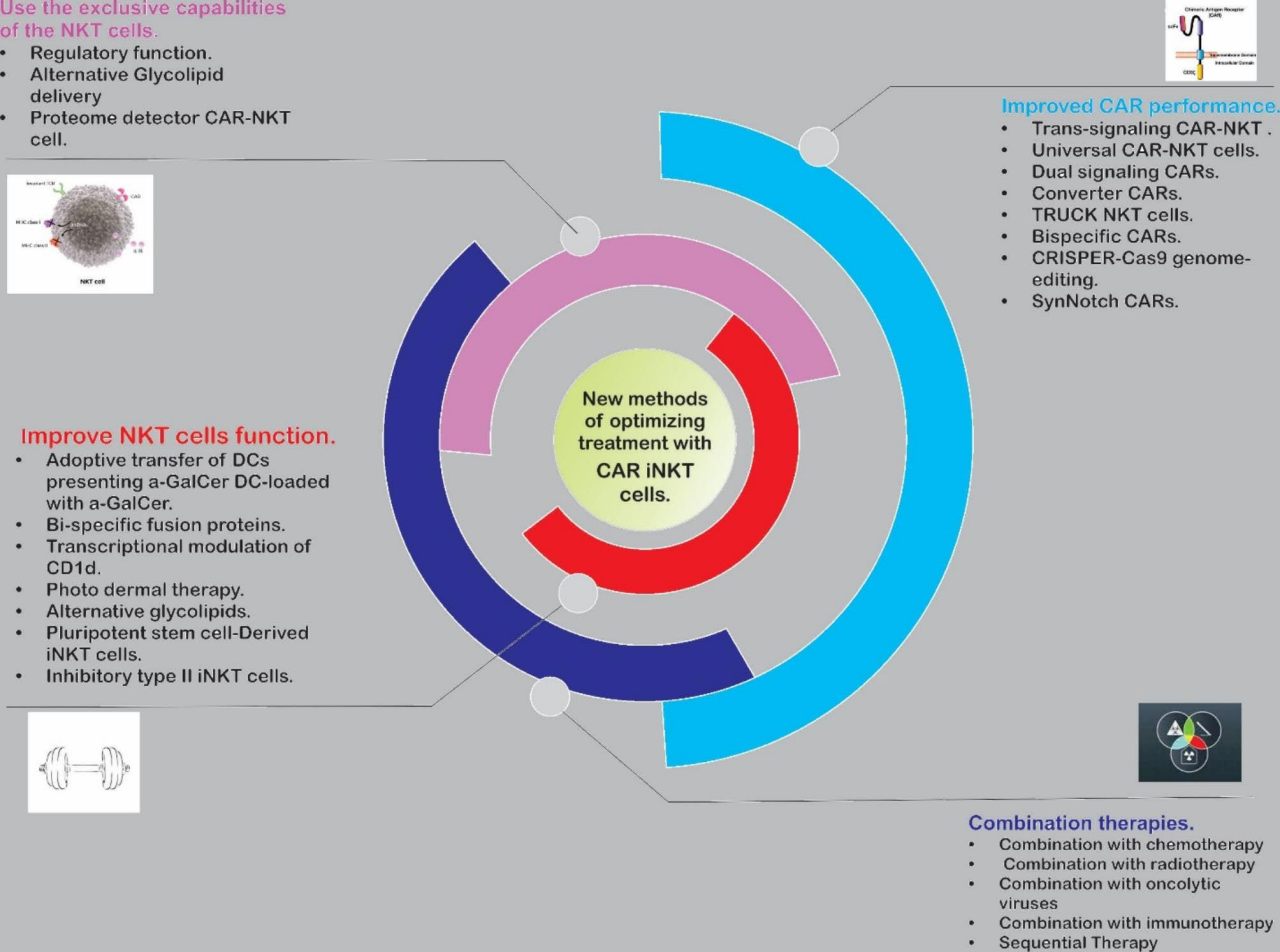
**The new methods of optimizing treatments with CAR-iNKT cells, overall overview.**

Like CAR-NK- and CAR-T-cells, CAR-NKT cell immunotherapy has some challenges that limit its function and broad usage in clinical settings *(*Fig. 3*)* [85, 100]. Encouragingly, some of these challenges are rectifiable by recently developed startegies like genome editing, designing smart-programmable CAR cells, and combinational therapy approaches [101–103]. All augmenting NKT cells are needed for the normal function cells to be more effective. This method is compulsory and the dysfunction of these cells, especially in IFN-γ production, reduces the quality of NKT cell-based immunotherapy in TME [104]. Another obstacle to the progress of NKT cell treatment is the low number of these cells at their source. Therefore, the progression in isolation and selective expansion of NKT cells can open new windows to NKT cell-based immunotherapy. These two recent problems have become very substantial crises in cancer patients because of their low number of immune cells, dysfunction, and low infiltration of immune cells into TME [105, 106]. Other hindrances include reduced expression of CD1d in tumor cells [107], the post-infusion α-GalCer anergy [20], and the long time of process and differentiation of NKT- and autologous DC-cells in a culture medium for adoptive transfer that causes some patients to give up their disease or no longer be eligible for treatment [79].

Compared to the normal microenvironment, the TME has differences in cells and factors like the type of APCs, antigen variants, cytokines, and metabolites that affect the anti-tumor functions of CAR-immune cells . In addition, TME has a limitation of glucose sources and lactate accumulation due to the glycolysis activity of tumor cells, which directly interferes with CAR-NKT cell function. Moreover, lack of glucose decreases IFN-γ production by interfering with TCR signaling, because glycolysis amplifies TCR vesicle recycling and maintains TCR signaling in NKT cells. Additionally, IL-4 production has the least negligible dependence on TCR signaling. Then, IL-4 production increases and polarizes cells toward Th2 cells,  which are considered a great favorite for tumor cells [104, 108].

Furthermore, the accumulation of lactic acid in TME inhibits the production of IFN-γ from NKT cells by interfering with lipid synthesis. Lactic acid reduces PPARγ expression in NKT cells by inhibiting the activation of mTORC1. In cooperation with PLFZ, the main transcriptional factor in NKT cells, PPARγ, promotes the SREBP1 transcription that controls lipid synthesis. Cholesterol improves TCR signaling and, as a result, increases the IFN-γ production. It has been reported that the reduction in PPARγ expression due to lactic acid accumulation in TME inhibited cholesterol synthesis and reduced the IFN-γ production by NKT cells in hepatocellular carcinoma of mouse models [50, 104, 109]. Disturbance in the crosstalk process between NKT cells and DCs leads to dysfunction in anti-tumor immune responses in TME. Crosstalk between these cells improves Th1 cell immune responses, which is mediated under the control of cell division control protein 42 homolog (Cdc42). The Cdc42 significantly controls cell migration and interaction, which decreased expression of Cdc42 in NKT cells and destroyed the intratumoral interaction and activation of NKT cells [50, 110]. The last challenge facing the advancement of CAR-NKT cell therapy is persistence duration in TME. As the tumor progresses, the function of the cells decreases significantly, and they become dysfunctional. Despite the broad efforts and breakthroughs in improving the CAR NKT-cell therapy in solid tumors, this treatment encounters some barriers like other therapeutic approaches. The available problems are common among other immunotherapy methods; some can be handled. It’s worth noting that CAR NKT-cell therapy challenges are very low compared to CAR T- or CAR NK- cell therapies.

## Safety and efficacy imrovemment strategies

### NKT cell innate feature reinforcement strategies

#### Regulatory function of CAR-NKT cells

The NKT cells have distinctive regulatory characteristics that may be more important than cytotoxic properties. The NKT cells can affect DCs through CD1d/TCR and CD40/CD40L interactions. They can also increase the expression of costimulatory molecules on DCs like CD40, CD80, and CD86 that induce IL-12 and CXCL16 production [111]. On the one hand, CXCL16 improves the IFN-γ production from NKT cells. On the other hand, IL-12 enhances IFN-γ production from NKT-, NK-, Th1-, and CD8^+ ^T-cells. This action constitutes a positive loop for supporting the anti-tumor activity of effector cells [43]. The NKT cells reprogram the polarization of M2 macrophage (tumor-associated macrophages or TAMs) to inflammatory M1 macrophages and decrease immunosuppression-dependent TME [112]. The accumulation of MDSCs in TME induces anergy in immune cells, disturbs immune responses, increases tumor cell growth, remodels TME, and elevates the metastasis rate. Interestingly, the NKT cells inhibit MDSCs by several mechanisms; for instance, they obstruct arginase-1 and NOS2 in influenza models [48], decrease the frequency and restrain the activity of MDSCs in 4T1 breast cancer models [113], and transduce α-GalCer loaded MDCSs to mature APC with the ability to induce the immune response in T- and NK-cells [114]. Furthermore, activated NKT cells reduce the number and function of type II NKT cells by the cross-regulation procedure and remove the harmful effect of these cells on the immune system, especially in cancer treatment [23, 115].

In addition to the self-regulatory function of NKT cells, these cells are regulated by other immune cells. For example, T_reg_ cells suppress the function of NKT cell-to-cell connection mediated by impaired DC maturation. T_reg_ cells affect CD4^+^ NKT cells more than CD4^−^ NKT cells and induce anergy in them that, in the end, decrease the clinical efficacy of NKT cell-based immunotherapy. So, T_reg_ cell depletion and NKT cell rehabilitation from the anergy state can increase NKT cell cytotoxic function in advanced cancer treatments [116]. The NKT cells can directly promote CD8^+^ T cell activation with several mechanisms, like CD1d interference, mediated by local soluble factors, mainly IFN-γ, direct interaction by T cells, and independent DCs [117]. Also, NKT cells can regulate the other compartment of the innate immune system with different mechanisms like cytokine secretion and direct interaction with other cells like NK cells [118]. As a result, the CAR-NKT cell has regulatory functions in almost all immune systems in addition to its cytotoxic ability. This effect plays a prominent role in cancer treatment due to the significant dysfunction in the immune system caused by cancer.

#### Alternative glycolipid delivery

Due to the different obstacles faced by α-GalCer infusion, getting help from vectors like artificial APCs, nanoparticles, exosomes, and liposomes may be helpful. Some studies have shown that compared to free α-GalCer, α-GalCer bounded vectors had beneficial effects in cancer treatment like increasing NKT cell expansion and cytokine production as well as decreasing the tumor burden [119–121]. This therapeutic effect can be increased due to enhanced uptake and presentation of antigens by DCs, which increments the downstream responses of NK- and T-cells [122]. Interestingly, α-GalCer-bonded vectors did not induce an anergy phenotype in NKT cells due to the reduced number of presenting α-GalCer B cells [123, 124]. Employing other vectors such as lipopolyplex vectors, silica microparticles, and poly lactic-co-glycolic acid (PLGA) nanoparticles also indicated enhanced NKT cell functions [125]. The critical point in utilizing vectors is whether these mechanisms retain their therapeutic advantage compared to α-GalCer in helping to improve the immune system [126]. The newly developed approaches led to improvements in drug or glycolipid delivery systems because their purpose is to packag the NKT cell activating factors in nanoparticle-based constructs. An important example of advanced immunotherapy is utilizing “associated agents” such as α-GalCer packaged into microspheres or liposomes that showed better functional response in cancer treatment as compared to CAR-NKT cell monotherapy [123, 127, 128].

#### Modified glycolipid analogs

Although α-GalCer is not faultless, almost all clinical trials on NKT cell immunotherapy used α-GalCer, as an NKT cell stimulator factor, highlighting the importance of α-GalCer. Encouragingly, a list of modified glycolipids is growing that might improve treatment effects. Chemical modification of α-GalCer leads to the production of several analogs of this ligand with a substantial effect on Th1 cytokine responses in NKT cells. For instance, replacing O-glycoside linkage with C-glycoside produces the α-c-GalCer structure with a high ability to increase the IFN-γ and IL-12 production from NKT cells [129, 130]. In addition, using α-c-GalCer does not escalate IL-4 production, which shows a skewing toward the Th1 phenotype [131]. Although α-c-GalCer has a better treatment effect than α-GalCer in models of melanoma, this substance could not stimulate human NKT cells successfully [130, 132]. With promising outcomes in preclinical fields, this substance creates hope for finding a new therapeutic agent with a decisive function in human NKT cells. The other substance is 7DW8-5, which has a stronger binding affinity to CD1d and TCR and is more effective at increasing IFN-γ and IL-2 production from NKT cells compared to α-GalCer [133]. The stimulation of NKT cells by 7DW8-5 showed raised Th1- and CD8^+^ T-cell responses in several mice-vaccinated cases, but it has not yet been used in cancer cases [134, 135]. Unlike α-c-GalCer, 7DW8-5 created more stimulation of human NKT cells in vitro [136]. Also, 7DW8-5 stimulated NKT cells in rhesus mascaques in influenza vaccines; because of the similarity of rhesus mascaques NKT cells to humans, 7DW8-5 maybe has an excellent potential to be investigated in human clinical trials as a therapeutic agent in the future [137].

Recently, a novel antigen glycolipid, called RK, has been developed to improve cancer treatment. According to published results, RK-loaded DCs improved the production of IFN-γ and T-cell long-term memory in both human and mouse models of cancer by enhancing NKT cell function [138, 139]. For example, it was found that the RK-loaded DCs applied in mouse models of metastasizing melanoma could prevent metastasis [139]. Although treatment with RK-loaded DCs was more effective than α-GalCer and had no adverse effects, their use in clinical trials needs more evidence [126]. The glycolipids may be helpful for effective CAR-NKT cell immunotherapy, but choosing the best of them requires more investigation.

### CAR-NKT cell performance improvement strategies

#### Transcriptional modulation of CD1d

One of the useful ways to amplify the performance of CAR-NKT cell therapy is by modifying target cells by transcriptional or epigenetic modulation of CD1d. For example, the anti-lymphoma function of CAR-NKT cells can be improved by inducing more translation and expression of CD1d on lymphoma cells and CLL-B cells, which is mediated by all-trans retinoic acid (ATRA). This platform can increase the function of NKT cell immunotherapy at the base of the CAR structure [12, 105].

#### Inverted cytokine receptors (ICR)/SynNotch CARs

Inverted cytokine receptors (ICR) and SynNotch CAR are synthetic transmembrane receptors activated by the link between tissue-specific ligands and cell surfaces [140, 141]. Activation of SynNotch receptors started the transcriptional pathways in the cell. After that, these receptors can improve the delicacy of tumor recognition by CAR-NKT cells and enhance their functions. This method has been evaluated in CAR-T cells and has had successful results, but it has not yet been used in CAR-NKT cells [142]. In addition, this method can be combined with other enhancement methods like TRUCK cells to intensify the efficacy of CAR-NKT cell therapy [143].

#### CRISPR-Cas9 genome-editing

Genome-editing (like CRISPR-Cas9), gene knockout, and gene-knockdown [like short hairpin RNAs (shRNAs)] technologies, are the other contributory approaches that have been developed to improve the anti-tumor function of CAR immune cells [144]. CRISPR-Cas9 is a gene-editing method with high on-target specificity for providing tumor-induced exhaustion cell resistance [145]. This technique has been used to delete the expression of the TCRα chain, TCRβ chain, β2M, and PD-1 in CAR-T cells [144, 146]. These techniques could affect stem cells by creating stable and impressive gene ablation in stem cell-differentiated immune cells [147]. Empowering the CRISPR/Cas9 system in the CAR-NKT cells is very limited, but the studies demonstrated its usefulness. For example, the first experience showed that using the CRISPR/Cas9 system in CAR-NKT cells could increase the persistency and the CD62-L expression [148]. In another study, overexpression of lymphoid enhancer-binding factor 1 (LEF1) by the CRISPR/Cas9 system in GD2-CAR NKT cells showed superior control in neuroblastoma xenograft mouse models [149].

#### Suicide-gene technology

Another approach is suicide gene modification which is combined with CAR-based treatment and prevents insertional oncogenesis risks [150]. Most viruses accepted for suicide genes come from herpes simplex thymidine kinase (HSV-TK), which showed sensation to the antiviral nucleoside analog ganciclovir (GCV) and provided the possibility of visualization by positron emission tomography (PET) for modified cell tracking [151]. Moreover, an alternative suicide switch system has been developed to utilize in CAR-NKT cell therapy and improve the treatment quality. Examples of these systems are fusion proteins expressing Fas signaling moieties [152] and caspase (icasp) under the post-transcriptional regulation of small molecule ligands [153].

#### TRUCK NKT cells

T-cell Redirect Towards Universal Cytokine Killing (TRUCK) is an engineered T cell with a transgenic payload that allows cell-producing proteins and other cell compartments by activating CAR [154]. TRUCK was engineered especially to improve the treatment function of CAR-T cells against solid tumors [155]. The most volatile component produced by TRUCKs is IL-12, which reprograms the TME and increases the anti-tumor function of CAR-T cells [155]. This method was applied to CAR-NK cells and had promising outcomes in lymphoma mouse models [89]. Hence, CAR-NKT cells would be appropriate candidates for transducing transgenic encoding NKT cell-stimulating cytokines such as IL-21 and IL-2 [156].

#### Proteome detector CAR-NKT cells (TCR-CARs)

NKT cells have an antigen recognition system independent of MHC-I, while the main focus in T cells is based on T-cell receptors (TCRs) [157]. Engineering the NKT cell based on TCRs leads to the MHC-I recognition ability that increases the treatment specificity of these cells. This option is used for NK cells. In this way, NK-92 cells are armored by TCR-CAR, which can recognize MHC-I molecules and significantly affect tumor cells [89]. Conversely, NKT cells have receptors for CD1d ligands expressed on target cells. Of this fact, the armored cells are created to target several tumor antigens that may indicate high-quality cancer treatment [158]. Although the effectiveness of this approach is still being proven, various investigations are required to obtain more information on whether it will destroy a considerable number of tumor cells.

#### Trans-signaling CAR-NKT cells (inducible CARs)

Inducible CARs have been recently added to the types of CARs. Inducible CARs have activation capacity after administering the drug to the body. This method has been tried in CAR-T cell immunotherapy, which subsequbtly increased IFN-γ production, cytotoxic function, and survival in mouse models [159]. Furthermore, proliferation and cytokine production are efficiently regulated by this system [160]. Interestingly, this method can prevent some side effects and improve the quality of treatment in routine immunotherapies. For example, inducible MYD88/CD40 (iMC), as a transcellular switch protein, that is activated after exposure to rimiducid, can be used in the inducible CAR system. iMC activation can induce the proliferation and activation of T cells, as well. A study evaluated the effect of ectopic IL-15.iMC on cytotoxicity and cytokine secretion in anti-CD123/BMCA CAR-NK cells. As a result, this structure enhanced the anti-tumor function and persistence of CAR-NK cell [161]. Hence, employing this practical strategy in the CAR-NKT cell structure would improve its anti-tumor activity.

### Advancement of the NKT cell function in CAR-NKT cell structure

#### DC-loaded α-GalCer; a new α-GalCer administration approach

Activating NKT cells with α-GalCer may be suitable but have different problems. Autologous NKT cells are suggested to proliferate *in vitro* with the α-GalCer substance before reinfusion to change the impairment of NKT cell function in TME [162]. For reducing the harmful effects of α-GalCer administration, such as induced anergy and low immunogenicity, using DCs loaded with α-GalCer has been suggested. Due to co-signaling by CD-40 and IL-21, DCs activate more NKT cells and have low-induced anergy [163]. This method decreased tumor cell growth and metastasis progression in tumor models [164]. It also increased the expansion and activation of NKT cells as well as IFN-γ production [92] without any particular side effects [165], primarily when using mature DC cells [166]. Besides, this strategy exhibited long survival in clinical trials and showed improved treatment outcomes in non-small cell lung cancer (NSCLC) patients [167]. This process does not have adverse effects when NKT cells are defective (like in different cancers) [126].

#### Bi-specific fusion proteins

The specificity of NKT cells can be increased by using α-GalCer/CD1d anti-tumor fusion proteins. One of the important factors that helps NKT cells affect tumor cells firmly is the existence of CD1d receptors on the tumor cells [107]. So, to strengthen the NKT cell function, the CD1d-antibody fusion protein can be applied to direct NKT cells toward tumor cells. In this manner, the N-terminus side of the CD1d ligand connects to B_2_-microglobulin and creates soluble CD1d (sCD1d). Then, an ScFv fragment connects to sCD1d against the tumor marker. This structure is loaded with α-GalCer before administration [168]. Recently, CD1d-antibody fusion proteins were made against tumor antigens like human epidermal growth factor receptor 2 (HER2), carcinoembryonic antigen (CEA), and CD19 [169, 170]. In this case, Horn et al. demonstrated that CD3xpd-L1 Bispecific T cell engagers (BiTEs) could activate T- and NKT-cells to destroy PD-L1^+^ tumor cells *in vitro* [171]. So, increasing the specificity of NKT cells maybe isn’t the most promising way to increase the effectiveness of NKT cell-based immunotherapy. This manner was also investigated in melanoma models, which improved the function of DCs, NK-, and CD8 T-cells [168]. When NKT cells are activated more than usual, they might induce tolerability of innate and adaptive cells and amplify immunosuppressive states in an anti-tumor manner [172].

#### Photothermal therapy

New research showed that photothermal therapy improved the anti-tumor function of NKT cells. This strategy uses several ways to create the conditions for the better function of NKT- and CAR-NKT-cells. Examples of these ways include increasing the blood flow in the tumor site and more secretion of cytokines and chemokines that lead to recruiting the immune cells to the tumor site, making an inflammatory condition, elevating tumor infiltration NKT-cells, and causing a higher secretion of IL-1 and IL-12 cytokines [173]. Production of these cytokines activates NKT cells and improves the recruitment and maturation of DCs, which positively affects the NKT cells [174]. In this case, the abovementioned mechanisms significantly boost the production of IFN-γ, CD107a, and granzyme B from NKT cells. These processes improve NKT cell function in a CD1d-dependent manner without any noticeable side effects [175].

#### Pluripotent stem cell-derived iNKT cells

One of the main obstacles to developing NKT cell-based immunotherapy is isolating adequate NKT cells from the PBMCs of cancer patients. Therby, suitable sources can help advance this treatment. One of the valuable strategies is using induced pluripotent stem cells (iPSCs) derived from patients [176]. Stem cells are good and acceptable sources for producing all immune cells and can accept genetic sequences to express them, like CARs. These cells have a self-renewing potential, which might decrease the need for several injections. Also, they can be used in an off-the-shelf process that can reduce the costs of immunotherapy. In addition, the genetic modification of these cells by novel tools, cell-arming, and the achievement of new anti-tumor mechanisms make them powerful cells against tumors. iPSCs-iNKT cells are generated from C57BL/NKT spleen cells, entering embryonic stem cells by nuclear transferring, and modifying by expressing reprogramming factors like Oct3/4, Sox2, KIf4, and Nanog to induce pluripotency with retroviral viruses [177]. Following the transfer to mice, the iPSCs-iNKT cells can generate a high amount of IFN-γ with maintained ability in the anti-tumor property. Additionally, several studies used iPSCs-iNKT cells and demonstrated high ability in cytokine production, anti-tumor cytotoxicity, and autologous NK cells upon activation by ligand-pulsed DC [31, 178]. Hence, the anti-tumor function would be more consolidated by creating iPSC-CAR NKT cells.

#### Inhibitory type II NKT cells

Type II NKT cells have a contributory function in tumors that escape from the immune system, which increases tumor survival. So, the restraint of these cells can be a helpful strategy for cancer immunotherapy. One of the approaches for preventing type II NKT cells is to apply lipids that change or block the responses of these cells. The NKT cells have several sulfatide antigens with different cell signaling functions. Recently, a unique sulfatide antigen isoform of type II NKT cells, namely C24:2, has been introduced and has shown significant inhibitory effects on the progression of lung cancer metastasis. In contrast, other types of sulfatide isoforms, such as C24:1 and C24:0, increased the development of tumor metastasis in mouse models of lung cancer [179]. CpG ODN is an agonist of TLR9 that has been used as a cancer vaccine adjuvant [180]. CpG ODN-activated NKT cells were shown to be functional in B16 melanoma models. Indeed, because DC cells were sensitive to this substance, they induced IFN-γ production from NKT cells without increasing the IL-4 level [181]. However, some studies found that CpG ODN limited NKT cells and showed regulatory function and negative feedback mechanisms in NKT cells that control NKT cell overactivated responses [22].

### Combination therapies with CAR-NKT cells

#### Combination with chemotherapy

Some chemotherapy drugs showed an immune-stimulating function, enhanced immune cell infiltration into TME, and increased effects of ongoing immunotherapy [182]. Utilizing chemotherapeutic agents like cisplatin, doxorubicin, methotrexate, and etoposide sensitizes tumor cells to NKT cells and affects TRAIL and Fas ligand (FasL) functions *in vitro* [183]. Besides, pretreatment with gemcitabine or cyclophosphamide induced-immunogenic cell death (ICD), raised the efficacy of NKT cell immunotherapy, and prolonged the survival in 4T1 breast cancer models *in vivo*. However, this increase was not seen in NK- and CD8 T-cell-based immunotherapies [184]. Those categories of drugs without the potential to induce ICD, like cisplatin and 5-Fluorouracil (5-FU), increase NKT cell function, tumor antigen release by dying cells, and antigen presentation by DCs, which were documented in mesothelioma and colon cancer models [185]. Among these chemotherapy drugs, lenalidomide, a multiplier cyclin-dependent kinase inhibitor, has had the best clinical results and could reduce tumor cell proliferation and cancer progression [186]. The effects of this drug include the reduction of vascular endothelial growth factor (VEGF) expression, restriction of tumor angiogenesis [187], augmentation of T cell proliferation, improvement of NK cell functions [188], enhancement of expansion of NKT cells, and upregulation of the IFN-γ production *in vivo* and *in vitro* [189]. Clinical findings in the myelodysplastic patients treated with lenalidomide indicated the abovementioned outcomes *in vivo* and *in vitro* [190]. In a phase I clinical trial study that used lenalidomide in combination with NKT cells in asymptomatic myeloma, the treatment was utterly tolerated by patients, except in one case that had an accompanying grade 3 adverse event. Treatment increased NKT-, NK-, monocyte-, and eosinophil-cells, which improved innate immune responses in tumor context. Also, this treatment reduced tumor-associated immunoglobulin in all patients except one, indicating a decrease in a tumor burden [165]. As a result, combining chemotherapy with CAR-NKT cell therapy is a perfect method to increase treatment efficacy, as the previous studies showed.

#### Combination with radiotherapy

Radiotherapy (RT) combined with cell-based immunotherapy has a beneficial effect on cancers. For example, the combination of RT with CAR-T cell therapy revealed encouraging outcomes in different cancers like B-NHL, lung cancer, and prostate cancer [191–194]. Recently, this strategy might be beneficial for treating CAR-T cell resistance solid tumors [195]. Regarding the CAR-NKT cell therapy, RT appeared to have high proliferation responses in NKT cells and improved their anti-tumor activity *in vitro*. Furthermore, the NKT cells showed better results along with RT than CD8^+^ T cells [196]. Inconsistently, the findings of a study indicated that the number of NKT cells and power of expansion in response to IL-2 and α-GalCer did not differ before and after the RT [197]. This issue has never been evaluated on CAR-NKT cells, and deciding on its effect requires more experiments on these cells. RT as a potent cancer treatment method would be a good therapeutic candidate for combining with CAR-NKT cells.

#### Combination with oncolytic viruses

Oncolytic viruses infect and lyse the tumor cells exquisitely [198]. The shreds of evidence showed that these viruses destroyed tumor cells directly and stimulated the immune system responses by increasing the activation of immune cells and more antigen presentation by APCs [199, 200]. Interestingly, it has been reported that combining NKT cell therapy with oncolytic viruses that process the high potential of ICD increases the quality of immunotherapy [201]. Examples of suggested oncolytic viruses for combining with the CAR-NKT cells to improve their function are vesicular stomatitis virus (VSV) and reovirus, which showed successful results. Accordingly, applying VSV along with NKT cell therapy improved survival and decreased the metastatic burden in breast and ovarian cancer mouse models compared to NKT cell monotherapy [200]. Moreover, the study demonstrated that the vaccine virus expressing 15-hydroxy prostaglandin dehydrogenase prostaglandin E2- inactivating enzyme, could sensitize the resistant tumors to iNKT cell treatment [202]. As a result, this combination therapy is another way to use CAR-NKT cells that had significant consequences in the studies.

#### Combination with checkpoint inhibitors

One of the helpful immunotherapy methods is exploiting the immune checkpoint inhibitors [203]. Combining NKT cell therapy with an immune checkpoint inhibitor suppresses inhibitory immune signaling and receptors. PD-1 and cytotoxic T-lymphocyte antigen-4 (CTLA-4) receptors inhibit the anti-tumor function of immune cells by blocking CD3/CD28 signaling, which is mediated by the up-regulation of glucose metabolism and interaction with intracellular pathways [204, 205]. CTLA-4 inhibitors are important in regulating immune responses and may be helpful in combination with NKT cell treatment [206]. The stimulation of α-GalCer and checkpoint inhibitors blocking PD-1 or PD-L1 can suppress the PD-1/PD-L1 axis and maybe help to overcome this limitation [207]. It was shown that in PD-1-resistant tumors, activation of NKT cells avoided CD8^+^ T cell anergy [208]. To this fact, a clinical trial study has been conducted to evaluate NKT cell therapy in combination with PD1/PDL1 inhibitors. In this trial, ABX196, a synthetically modified α-GalCer activating NKT cells, was used, which resulted in elevated anti-tumor cytotoxicity in Hepa 106 xenograft hepatocellular carcinoma (HCC) models (NCT03897543). Administration of ABX196 increased IFN-γ production by NKT cells and Th1 skewing without affecting the IL-4 production in TME. ABX196 boosted tumor regression and increased survival when used alone or in combination with anti-PD-1 in cancer models of melanoma, colon carcinoma, and breast [209–211]. This trial evaluated ten patients, and clinical benefits were seen in five of them. One patient had a partial response, and four patients had stable disease without any life-threatening side effects. As a result, ABX196, in combination with a PD-1 blocking antibody (Nivolumab), had a significant effect on HCC patients with previous immune checkpoint inhibitor (ICI) therapy [212]. Recruiting checkpoint inhibitors as a novel immunotherapy method can effectively influence CAR-NKT cell therapy and improve patient clinical outcomes.

#### Combination with immunostimulators

Another proper immunotherapy strategy for combining with CAR-NKT cell therapy is using immunostimulatory agents such as monoclonal antibodies (mAbs) and cytokines [126]. The mAbs target immunostimulatory receptors like 4-1BB, CR5, and CD40, then activate anti-tumor immunity that affects many cancers [213]. The different mAbs, such as anti-CD52, anti-VEGF, anti-CD33, anti-HER2, and anti-EGFR, have been used in the cancer treatment and showed significant improvement outcomes. In spite of the clinical benefits, some of them showed limited responses, different side effects, and cancer relapse [214]. However, research documented that α-GalCer combined with agonistic anti-DR5 and anti-4-1BB antibodies or agonistic anti-CD40 and anti-4-1BB antibodies significantly led to tumor regression, and about 80% of mice underwent complete regression [215, 216]. In the melanoma and breast cancer mouse models, treatment with α-GalCer and immunostimulator cytokines, like IL-12 and IL-21, begets tumor regression and increased survival [217]. Unfortunately, few studies worked in this manner due to concerns about the safety of immunostimulatory antibodies and effective results in human clinical trials, so for particular decisions, more investigations are required [218].

One of the other solutions to improving cancer treatment is the enhancement function of NKT cells in TME and better recognition of Th1-based lipid antigens [110]. For example, IL-15 co-expression in CAR-NKT cells increases their localization at the tumor site and improves tumor control without any toxic effects. Furthermore, the IL-15 cytokine has been studied for its ability to improve anti-tumor function. IL-15 has a critical role in the function of T- and NKT-cells and ameliorates the tumor-cell killing activity of CAR-NKT cells. As mentioned previously, one of the crucial challenges in CAR-NKT cell therapy is the survival and quality of these cells in the TME or PBMCs. The combination of different immunostimulatory agents can enhance cell conditions in emergency environments. It has been suggested that the IL-15-conjugated CD28-CAR structure reduces exhaustion markers, upgrades persistence, and enhances the anti-tumor function of CAR-NKT cells [219]. GD2-CAR IL-15 NKT cells could improve cell persistence in vitro, augment cell localization in tumor sites, and enhance tumor control compared to GD2-CAR NKT cells in mice bearing neuroblastoma. Notably, IL-15-conjugated GD2 CAR NKT cells reduced the expression of PD-1 receptors [63]. These characteristics of IL-15 make it an excellent candidate for designing the different types of CAR-NKT cells based on utilizing potent cytokines against cancer. It has been reported that the activation of PPARγ with its agonists could improve the anti-tumor responses and superior tumor control along with IFN-γ production from tumor-infiltrated NKT cells [63, 104]. So, involving immunostimulator factors like potent cytokines or mAbs in CAR-NKT cell therapy enhances treatment efficacy and helps create promising results.

## Conclusion and future perspective

Due to the complexity, smarts, and various escape routes of tumors from the immune system, studies should focus on developing and recruiting intelligence-based treatments like immune-based therapies. The outstanding cytotoxic and regulatory characteristics of CAR-NKT cells with functional capabilities are considered a potent newfound cell-based therapy against cancers. This treatment is entirely novel, and reaching the definitive treatment has a long way to go. CAR-NKT cells possess the most properties of both T- and NK-cells, making them powerful and appropriate cell-based therapeutic choices against cancers. In addition, the CAR-NKT cells have different anti-cancer abilities, such as rapid recognition of the tumor cells, a higher rate of infiltration into TME, promising cytotoxicity, and regulatory function on immune cells. By focusing on past experiences in CAR T- and CAR NK-cell therapy, engineering the intelligent and effective CAR-NKT cell structure and combining it with other therapeutic strategies may open new windows in cancer treatment. The preclinical studies demonstrated noticeable and promising findings of CAR-NKT cell therapy in various cancers, and scientists hoped they would achieve a successful treatment. However, unfortunately, the limited number of clinical trial studies developed or in progress and the lack of access to more definitive results have made the questionable and controversial decision regarding this potent treatment strategy. Utilizing the new contributory solutions such as engineering armored and programmable CARs, progress in gene transfer strategies, and improving the immune system situation in various ways can help achieve potent CAR-NKT cell therapy. The interaction between the immune system and genetics can create new and intelligent treatments that we can one day use to treat cancers. CAR-NKT cell therapy needs more studies and a bright future in cancer treatment, and there have been many experiences for us to continue.

## Data Availability

Not applicable.
